# Detection of residual and chemoresistant leukemic cells in an immune-competent mouse model of acute myeloid leukemia: Potential for unravelling their interactions with immunity

**DOI:** 10.1371/journal.pone.0267508

**Published:** 2022-04-29

**Authors:** Alexia Mopin, Frédéric Leprêtre, Shéhérazade Sebda, Céline Villenet, Meriem Ben Khoud, Martin Figeac, Bruno Quesnel, Carine Brinster

**Affiliations:** 1 Univ. Lille, CNRS, Inserm, CHU Lille, UMR9020-U1277 – CANTHER - Cancer Heterogeneity Plasticity and Resistance to Therapies, Lille, France; 2 Institut pour la Recherche sur le Cancer de Lille (IRCL), Lille, France; 3 Univ. Lille, UAR2014 - US 41 - Plateformes Lilloises en Biologie & Santé- Plateau de génomique fonctionnelle, Centre de biologie Pathologie Génétique - CHU Lille, Lille, France; All India Institute of Medical Sciences, INDIA

## Abstract

Acute myeloid leukemia (AML) is characterized by blocked differentiation and extensive proliferation of hematopoietic progenitors/precursors. Relapse is often observed after chemotherapy due to the presence of residual leukemic cells, which is also called minimal residual disease (MRD). Subclonal heterogeneity at diagnosis was found to be responsible for MRD after treatment. Patient xenograft mouse models are valuable tools for studying MRD after chemotherapy; however, the contribution of the immune system in these models is usually missing. To evaluate its role in leukemic persistence, we generated an immune-competent AML mouse model of persistence after chemotherapy treatment. We used well-characterized (phenotypically and genetically) subclones of the murine C1498 cell line stably expressing the *ZsGreen* reporter gene and the WT1 protein, a valuable antigen. Accordingly, these subclones were also selected due to their *in vitro* aracytidine (Ara-c) sensitivity. A combination of 3 subclones (expressing or not expressing WT1) was found to lead to prolonged mouse survival after Ara-c treatment (as long as 150 days). The presence of residual leukemic cells in the blood and BM of surviving mice indicated their persistence. Thus, a new mouse model that may offer insights into immune contributions to leukemic persistence was developed.

## Introduction

Acute myeloid leukemia (AML) is a malignancy that affects different differentiation stages of myeloid cells, leading to their dysfunction and clonal expansion in the bone marrow (BM) and peripheral blood (PB) of affected individuals [1]. Different genetic aberrations can be associated with this disease, such as fusion genes (translocations/inversions), mutations or WT1 (Wilm’s tumor 1) overexpression. Current intensive chemotherapy protocols (cycles of cytarabine and anthracycline) allow complete remission (CR) in 50 to 80% of AML patients depending on their age and the AML type involved [2]. However, relapses are often observed within 2 to 60 months posttreatment in 60 (young) to 80% (elderly) of patients. These relapses have been proposed to be due to the persistence of chemoresistant leukemic cells, also termed minimal/measurable residual disease (MRD). MRD can be monitored in the BM and PB of patients by flow cytometry and/or molecular biology techniques (RT-qPCR, next-generation sequencing) when AML is associated with genetic alterations. Several studies have demonstrated that subclonal heterogeneity and the existence of leukemia-initiating cells (LICs) (including leukemic “stem-like” cells (LSCs) and their subsequent progeny) may be responsible for relapses [1, 3–7]. Indeed, Bachas *et al*. showed that a minor subpopulation of LICs responsible for relapse was already present at diagnosis in patients [8]. Ding *et al*. employed whole-genome sequencing and analyzed that two major clonal evolution patterns of leukemic cells can occur following chemotherapy protocols: the emergence of 1) some minor subclones (carrying diagnosis/relapse mutations) or 2) founding subclones with additional specific mutations to escape chemotherapy [9]. Xenograft AML mouse models are based on the injection of patient-derived leukemic cells (PDXs) or leukemia cell lines in immune-deficient mice. They represent valuable tools to assess the presence of subclonal populations and LICs and test new therapeutic strategies [3, 4, 7, 10, 11]. However, the lack of an immune system in these mouse models raises questions about its contribution to the hierarchy, persistence or elimination of engrafted leukemic cells [1]. Indeed, T cells, macrophages and their released cytokines (interferon-gamma, tumor necrosis factor-alpha) were shown to contribute to cancer cell persistence through their growth control or induction of cellular quiescence/senescence in solid cancer mouse models [12–15]. In AML patients, whether the immune system plays a role in MRD remains unclear, but residual T lymphocytes, including regulatory T cells, were shown to subsist at high frequencies during and after chemotherapy, and faster lymphocyte reconstitution was associated with CR and increased AML survival [16–18]. *In vivo* cotransplantation of AML patient-derived T cells with leukemic cells (unfractionated blood samples) in immune-deficient mice led to GvHD (graft-versus-host disease) rather than AML disease [19]. Actual immune-competent AML mouse models are based on the transplantation of transduced cells with fusion genes (*RUNX1-ETO(9a)*, *CBFB-MYH11*, *MLL-AF9*) or their expression after transgenesis, conditional knock-in or chromosomal translocation. However, these models also present limitations in investigating the role of the immune system in leukemic cell lifespan and persistence due to their disease development latency (≥ 16 weeks), the subsequent difficulty associated with the kinetics of chemotherapy and the absence of information on subclonal populations [20–22].

Based on these observations, we decided to generate an immune-competent mouse model of leukemia MRD after injecting murine AML C1498-derived subclones. We previously showed that intravenous injection of these leukemic cells in syngeneic mice led to acute myelomonocytic leukemia in 17 to 19 days with features of the human pathology [23]. As observed for primary human AML cells at diagnosis, the C1498 murine AML cell line is also genetically heterogeneous and contains different subclones harboring common and specific genetic alterations [24]. Whether subclonal populations described in AML-affected patients possess intrinsic phenotypic and/or functional properties that would explain their chemotherapy resistance and persistence *in vivo* is not yet clear. Therefore, to generate this leukemia MRD mouse model, we used these different murine subclones and tested their chemotherapy sensitivity *in vitro* and *in vivo* using different cell doses, combinations and chemotherapy schedules. The *Wt1* gene was also overexpressed in these murine subclones, as observed in 70 to 90% of AML-affected patients. Indeed, *WT1* expression can be used in patients for MRD assessment and follow-up posttreatment when no other marker is available and also represents a valuable leukemia-associated antigen [25–28]. We established two new immune-competent mouse models of leukemia persistence (expressing or not expressing *Wt1*) that are useful to study the role of the immune response in the control of residual AML cell proliferation and survival and test new immunotherapeutic approaches.

## Materials and methods

### Mice and AML C1498-derived subclones

Four-week-old female C57BL/6J mice were purchased from Charles River (L’Arbresle, France). The mice were maintained under specific pathogen-free conditions and used between 5 and 6 weeks of age. Housing and all experimental procedures were approved by the French Ministry of National Education and Research (APAFIS#3813–2016012715139138) and performed in accordance with the French and European Guide for the Care and Use of Laboratory Animals. C1498 AML subclones stably expressing ZsGreen fluorescent protein were maintained in complete RPMI-1640 medium as previously reported [24]. We performed the exome of all subclones and 10 specific mutated genes were selected for each subclone. These genes regions were amplified by PCR and sequenced after thawing of frozen vials and before injection of the cells in mice to ensure for the presence of specific mutations for each subclone and the lack of clonal drift in *in vitro* cultures.

### Generation of WT1-expressing subclones after stable transfection

The mouse *Wt1* gene was amplified from the pCMV6-Wt1 (Myc-DDK-tagged) vector (Origene, Herford, Germany) and cloned into the pVITRO.1 plasmid (containing neomycin gene resistance) (Invivogen-Cayla, Toulouse, France) under the control of the mouse elongation factor-1-alpha promoter. The different subclones were transfected with the pVITRO.1/Wt1 plasmid by electroporation (Amaxa Systems-Lonza, Amboise, France) and selected for stable expression of the murine WT1 protein due to neomycin resistance. *Wt1* gene expression was determined for each subclone by RT-qPCR and normalized to *Abl1* gene expression. All subclones still presented their specific mutations after *Wt1* stable transfection.

### Detection of the WT1 protein by western blot analysis

Protein lysates were extracted from stably transfected WT1-expressing cells using M-PER^™^ Mammalian Protein Extraction buffer (Thermo Fisher Scientific, Waltham, MA) containing a protease inhibitor mix (Roche Diagnostics, Basel, Switzerland) and phosphatase inhibitor cocktails (B and C) (Santa Cruz Biotechnology, Dallas, TX) on ice for 10 min. After centrifugation at 14,000 g for 15 min at 4°C, the cellular supernatants were collected and quantified using a BCA protein assay kit (Pierce, Thermo Fisher Scientific). Thirty micrograms of protein extracts were separated on a 12% SDS-PAGE gel and transferred to nitrocellulose membranes (all from Invitrogen, Thermo Fisher Scientific). The membranes were blocked with 5% nonfat dried milk in TBS 0.2% Tween-20 for 1 h at room temperature and incubated overnight at 4°C with a specific primary anti-WT1 antibody. Membranes were washed 3 times in TBS 0.2% Tween-20 before adding the horseradish peroxidase-conjugated secondary antibody for incubation at room temperature for 2 h. Membranes were washed again 3 times in TBS 0.2% Tween-20. Development was performed using ECL plus detection reagent for 5 min and read using Image Quant LAS 4000 (GE Healthcare, Chicago, IL). Beta-actin reference protein staining was realized after stripping the membranes with Restore^™^ PLUS Western Blot Stripping solution (Thermo Fisher Scientific) for 5 min under gentle shaking at room temperature.

Beta-actin labeling was performed as indicated for WT1 staining using a primary anti-beta-actin antibody and a secondary antibody (all purchased from Santa Cruz Biotechnology).

### *In vitro* cell survival assessment with cytarabine

Subclone viability to cytarabine was evaluated using an MTT assay (Sigma-Aldrich, Lyon, France). A total of 4×10^4^ cells/well were seeded in a round-bottom 96-well plate in 80 μl of RPMI-1640 (without phenol red) supplemented with 10% FBS. Cytarabine solution was added in triplicate to each well with increasing concentrations from 0 to 100 μg/ml. The plates were then incubated at 37°C for 72 h. Subsequently, 10 μl of MTT (5 mg/ml in PBS) was added to each well and incubated for 4 h at 37°C, and then cells were lysed by the addition of 100 μl isopropranol/HCl solution (Sigma). Absorbance was evaluated at 570 nm using a SpectraMax^®^ microplate reader (Molecular Devices, San José, CA). Cell survival was calculated as the percentage of absorbance obtained for treated cells compared with that of nontreated control cells.

### Animal injections, cytarabine administration, survival follow-up and organ cell collection

This study was carried out in accordance with the recommendations of the French and European Guide for the Care and Use of Laboratory Animals. The protocol was approved by the local ethical committee followed by the French Ministry of National Education and Research (APAFIS#3813–2016012715139138). All procedures were designed to minimize the animals suffering. 10^5^ leukemic cells were injected into the tail vein (intravenous route, IV) of each mouse. Mice succumbed to AML development in an average of 31, 32 or 38 days depending on the injected subclone(s). Cytarabine was administered at different doses (100 or 200 mg/kg) intraperitoneally (IP) daily to each mouse. Mice were monitored for survival or euthanized whenever they presented the first signs of distress (isolation in the corner of the cage, slow moving, no more shiny fur) due to AML disease. A total of 224 mice were used in this study and, although their survival was monitored daily after AML cell injection, about 16% of the injected animals were found dead in the cage due to AML.

When injecting 3 subclones (C5, B11, E7) with or without WT1 expression, the mean number of *ZsGreen* copies/mouse at day 0 was estimated to be 521,343±210,812 per 10^4^ *Abl1* copies (the mean ± SEM from 6 independent experiments). Similarly, the mean number of *Wt1* copies injected/mouse at day 0 was estimated to be 21,038±/4,779/10^4^ *Abl1* (the mean ± SEM from 5 independent experiments).

Organs were harvested from mice and mechanically disrupted through a 70-μm strainer. Bone marrow (BM) cells were collected from tibias and femurs. After cutting bone extremities, they were flushed out using a 26-gauge needle either into ice-cold PBS for flow cytometry analysis or directly into RNAlater^®^ (Qiagen, Courtaboeuf, France) for RT-qPCR experiments. After red blood cell (RBC) lysis buffer was added (BD Biosciences, Le Pont de Claix, France), the organ cells were washed with cold PBS and resuspended in cold FACS buffer (0.5% BSA, 2mM EDTA, PBS) for flow cytometry staining. Peripheral blood samples were collected through the submandibular vein. RBCs were lysed using 1 ml of RBC buffer (310 mM NH_4_Cl, 20 μM Na_2_EDTA, 20 mM KHCO_3_) for less than 2 minutes, washed twice in PBS and resuspended in FACS buffer for analysis by flow cytometry.

### Flow cytometry

For ZsGreen fluorescent protein detection, blood samples and 10^6^ organ-derived or cultured cells were stained with 1 μg/ml propidium iodide to exclude dead cells, washed twice in FACS buffer and resuspended for analysis. Flow cytometry analysis was performed on a Cyan ADP analyzer using Summit 4.3 software.

### Reverse transcription and real-time polymerization chain reaction (RT-qPCR)

Total RNA was extracted from RBC-lysed blood cells or BM using the Nucleospin^®^ RNA kit (Macherey-Nagel, Hoerdt, France) and stored in RNAlater^®^ (Qiagen). Complementary DNA was generated with the Quantitect Reverse Transcription^®^ kit (Qiagen). The murine *Abl1* gene was used as a reference for normalization, and a commercial plasmid (pCMV6-Abl1, purchased from Origene) was serially diluted to generate a standard curve. Real-time PCR experiments were performed in triplicate or more per organ/mouse or cells, and 40 cycles of amplification were realized. Its analysis was performed using the Step One^®^ system and dedicated software (Applied Biosystems). DNA samples from organs were amplified for *Wt1* and *ZsGreen* gene expression and *Abl1* normalization using TaqMan^™^ predesigned or custom (for *ZsGreen*) primers/probe kits and Gene Expression Master mix (Applied Biosystems, Thermo Fischer Scientific). *ZsGreen* and *Abl1* genes were amplified from cells in the same run using the respective sense and reverse primers and the GoTaq^®^ qPCR Master Mix (Promega, Lyon, France): 5’-CCCGTGAAGACCGCAGCGAT-3’, 5’-CGACCGGCGCTCAGTTGGAA-3’ and 5’-ATGGCATGTCACCTTACCCG-3’, 5’- GTTCCACTGCCAACATGCTC-3’.

To determine sensitivity thresholds for *ZsGreen* and *Wt1* qPCR assays, 3 subclones mixture (C5, B11, E7) with or without WT1 was serially diluted in total BM cells. Total RNA isolation and RT-qPCR were performed as previously described. The detection thresholds in BM cells were defined as 1x10^-4^ for *ZsGreen* and 1x10^-3^ for *Wt1*.

### Targeted next-generation sequencing

A custom AmpliSeq HD panel was designed to cover specific variants of the B11, C5 and E7 subclones. The panel was designed for ThermoFisher sequencers on the Ampliseq Designer tool and was manufactured by Thermo Fisher.

### Library preparation

AmpliSeq libraries were prepared with the Ion AmpliSeq Library Kit 2.0, the Ion Xpress Barcode Adapter Kit, and primers. Ten nanograms of each DNA sample was used as a template to prepare the library according to the manufacturer’s instructions. Quality control and quantification of all libraries were performed on an Agilent 2200 (Agilent Technologies, Inc., Wilmington, Delaware) and Tapestation using the High Sensitivity D1000 ScreenTape assay. After normalization, the libraries were pooled. Emulsion polymerase chain reaction (PCR) was used to clonally amplify the obtained library pool, which was diluted to 65 μM. Emulsion PCR, enrichment, and chip loading were performed on an Ion Chef instrument with the Ion Kit-Chef (Thermo Fisher Scientific). Twenty-four barcoded samples were sequenced using Ion 530 chips on the Ion S5XL system (Thermo Fisher Scientific). Torrent Suite (V5.10.2), including coverage analysis (V5.10.0.3) and variant calling (V5.10.1.20), on the mm10 reference genome was applied with optimized settings for the HD option (Thermo Fisher Scientific).

### Immunofluorescence staining for microscopy

*In vitro* cultured subclones (untransfected or stably transfected with the pVITRO.1/Wt1 plasmid) were harvested, and 10^5^ cells were centrifuged on slides at 4.52 x g for 2 min. Cells were fixed using PFA 1% for 10 min and washed twice in PBS for 5 min. They were incubated twice in glycine 0.1 M for 10 min before being washed again in PBS twice. Cells were incubated in saturation/permeabilization buffer (SPB) (BSA 4%/Triton 0.1%) at room temperature (RT) for 1 hour. Staining was performed with a primary anti-Myc-tagged antibody (Sigma Aldrich, Saint Quentin Fallavier, France) (diluted 1:100 in SPB) for 2 hours at RT, and slides were washed 3 times in PBS for 5 min. An anti-mouse IgG coupled to AlexaFluor594 was used as the secondary antibody (Interchim, Montluçon, France) (diluted 1:200 in SPB) for 2 hours at room temperature. Slides were washed 3 times in PBS for 5 min before mounting in Vestashield^™^ medium (Thermo Fisher Scientific, France). Staining results were observed on a Leica DMI8 microscope (Leica Microsystems, Nanterre, France).

### Statistical analyses

Graphs and statistical analyses were generated using GraphPad Prism 5.0. Unpaired Student’s t-tests were used to compare IC50 values and different groups of mice (controls, AML-succumbing or AML-surviving mice). Mouse survival curves were compared using the log-rank test.

## Results

### C1498-derived subclones are sensitive to Ara-c and express high levels of the *ZsGreen* transcripts necessary for AML MRD detection

**Fig 1:**
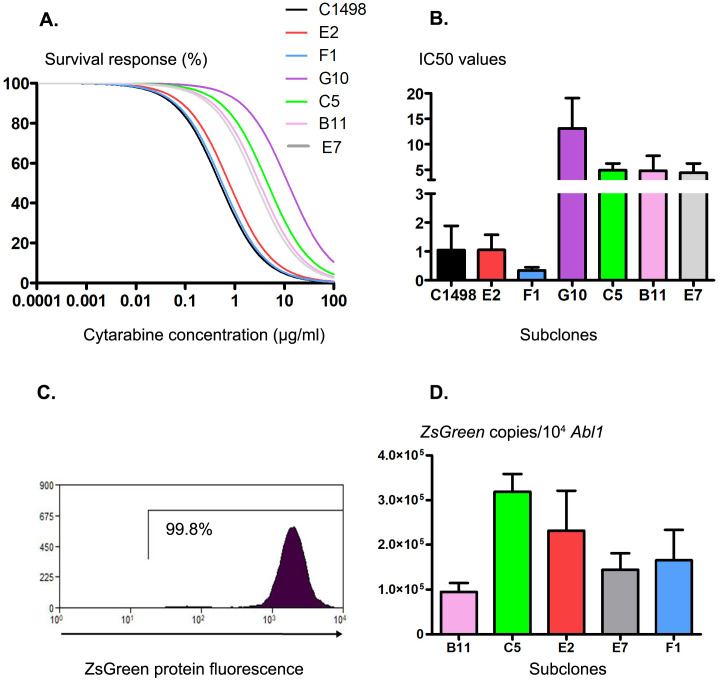
Ara-c sensitivity of the different leukemic *ZsGreen*-expressing subclones. (A) *In vitro* survival responses of the different subclones to increasing cytarabine concentrations and (B) representation of the cytarabine sensitivity of the different cell lines using IC50 values. The results are represented as the mean from 3 independent experiments for each subclone starting at cytarabine concentrations of 10^−4^ μg/ml (100% survival). (C) Representative flow cytometry histogram of ZsGreen protein expression in each subclone. (D) *ZsGreen* gene expression in each subclone determined by RT-qPCR. The number of *ZsGreen* transcript copies was normalized to 10^4^ copies of *Abl1* using a plasmid reference curve.

In a previous study using the murine C1498 AML cell line, 6 different subclones with genetic and immunologic heterogeneity were characterized [24]. For this study, we decided to use these well-defined subclones (phenotypes and presence of specific genotypic mutations) to generate a reproducible mouse model of leukemia MRD. In patients, conventional AML therapy includes cycles of cytarabine (Ara-C) and an anthracycline. As anthracycline (daunorubicin or doxorubicin) injections in mice (by intravenous or intraperitoneal routes) were shown to result in severe toxicity [22, 29], we chose Ara-c as the main chemotherapeutic agent. The 6 different subclones were tested for their Ara-c sensitivity *in vitro* and compared to the parental C1498 cell line (Fig 1A and 1B). All subclones were found to be sensitive to Ara-c with different mean IC50 values (Fig 1B). The parental cell line and the F1 and E2 subclones showed the highest sensitivity to Ara-c, with IC50 values ranging from 0.34±0.11 to 1.05±0.52, and the B11, C5 and E7 subclones had comparable mean IC50 values (4.82±2.90, 4.90±1.31 and 4.39±1.84, respectively). The G10 subclone presented a preeminent IC50 value (a mean of 13.12±5.93) 3 to 38 times higher than the other subclone mean IC50 values and was excluded from further experiments for this reason. The selected subclones stably overexpressed the fluorescent ZsGreen protein as previously described [24] (Fig 1C). This protein was shown to be poorly immunogenic *in vivo* [30] and its overexpression was used for leukemic cells’ detection and estimation in organs. Concomitantly, its gene expression was normalized to 10^4^ copies of the *Abl1* gene for AML cells’ assessment, as performed for genetic abnormality quantification for the diagnosis of AML disease in patients and their MRD follow-up in clinical practice [2]. *ZsGreen* expression ranged from 94,815±19,562 copies/10^4^ *Abl1* (948.1% *ZsGreen*/*Abl1*) for the B11 subclone to 318,875±39,666 copies/10^4^ *Abl1* for the C5 subclone (3,188.7% *ZsGreen*/*Abl1*) (Fig 1D). These values were found to be in the expression range of the most frequently encountered genetic aberrations described in peripheral blood or medullary blasts of AML-affected patients at diagnosis (*NPM1* mutations, ranging from 10 to 10,000% *NPM1*/*ABL1* or the *RUNX1/RUNX1T1* fusion gene, ranging from 100 to 1,000% *RUNX1/RUNX1T1*/*ABL1)* [26, 31, 32]. AML patients (70 to 90%) also overexpress the *Wt1* gene at diagnosis, and its expression is routinely used as an MRD marker post chemotherapy and as a predictor of relapse. The WT1 protein represents one of the best characterized antigens in AML disease and a suitable target for blast elimination. Thus, we also decided to generate *Wt1*-overexpressing subclones to establish our immune-competent leukemia MRD mouse model.

### WT1 protein expression in the subclones maintained their *ZsGreen* expression and sensitivity to cytarabine

**Fig 2:**
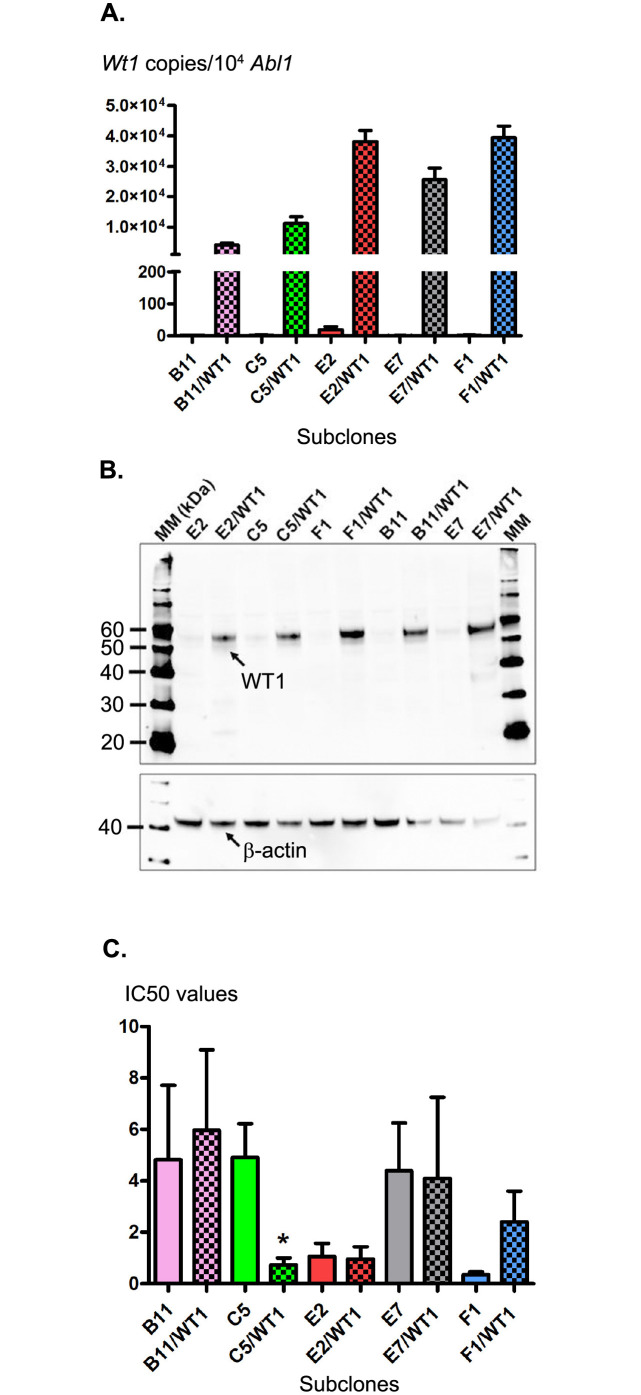
WT1 protein expression in different subclones. Different subclones were stably transfected with the pVITRO.1/Wt1 plasmid, and WT1 gene and protein expression levels were confirmed by RT-qPCR (A) and western blot (B), respectively. The subclones’ sensitivity to cytarabine treatment *in vitro* was also assessed and visualized using IC50 values for comparison with their respective untransfected subclones (C). For qPCR and survival assays, the mean ± SEM from three independent experiments are depicted.

We then stably transfected the 5 selected subclones to express the murine *Wt1* gene. Its expression was verified by RT-qPCR, and each subclone was selected for its highest expression, which was comparable to the overexpression level observed in AML-affected patients (5 to 1,000% *WT1*/*ABL1*) (Fig 2A) [26]. *Wt1* expression ranged from 4,123±656.8 copies/10^4^ *Abl1* (41% *Wt1*/*Abl1*) for the B11 subclone to 39,517±3,793 copies/10^4^ *Abl1* (395% *Wt1*/*Abl1*) for the F1 subclone. The presence of the most highly expressed intracellular WT1 proteins of 52 and 54 kDa (isoform D) was confirmed by western blotting in each selected subclone (Fig 2B) and by immunofluorescent staining (S1 Fig). ZsGreen protein expression remained unchanged when assessed by flow cytometry (S1 Fig) and RT-qPCR (S1 Fig). A survival assay with Ara-c was then performed to determine whether WT1 protein expression could interfere with sensitivity to the drug (Fig 2C). Only the C5/WT1 subclone was shown to become significantly more sensitive to chemotherapy (4.90±1.31 versus 0.73±0.26 with WT1 expression), and other subclones presented a comparable response to Ara-c.

### Ara-c treatment leads to significantly prolonged survival of AML-bearing mice when injected with 3 subclones expressing or not expressing the WT1 protein

**Fig 3:**
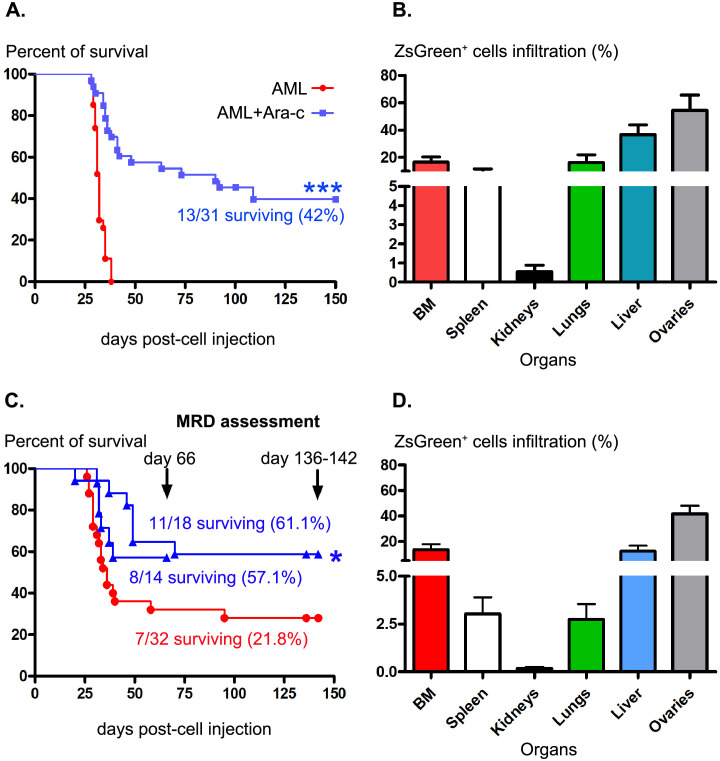
Ara-c treatment favors the prolonged survival of mice injected with B11/C5/E7 subclones expressing or not expressing WT1. (A) Survival curves of leukemic mice receiving (blue line) cytarabine treatment or not (red line) 10 days after joined injection of the B11, C5 and E7 subclones. (B) Percentages of ZSGREEN^+^ cell infiltration in lymphoid and nonlymphoid organs in B11/C5/E7 AML-succumbing mice. (C) Survival curves of animals injected with B11/C5/E7/WT1-expressing subclones and treated (blue line) or not (red line) with chemotherapy. Mice were sacrificed 66 and 136–142 days after cell injection for residual AML cell assessment. (D) Percentages of ZSGREEN^+^ cell infiltration in different organs in B11/C5/E7/WT1 AML-succumbing mice. ***, *p<0*.*0001*, log-rank test comparing B11/C5/E7-injected mice treated or not with Ara-c; * *p = 0*.*0174*, comparison of B11/C5/E7/WT1-bearing animals with or without chemotherapy treatment using the log-rank test and GraphPad Prism (V5.0).

To establish our AML MRD mouse model, we injected the different *ZsGreen*-expressing subclones under various conditions: injection of a unique subclone, a combination of 2 or 3 subclones or all subclones (combination of 5). The E2 subclone was injected alone because it presented fine Ara-c sensitivity (a mean IC50 value of 1.05±0.52) and elevated *ZsGreen* expression. For the same reasons, the E2 and F1 subclones were also injected together. The 3 other subclones, B11, C5 and E7, with lower but comparable Ara-c sensitivity (Figs 1A, 1B and 2C) were tested jointly. Injection of 10^5^ cells led to AML onset in 35 days on average, allowing administration of chemotherapy (Table 1). Different doses of Ara-C (3 to 5 injections of 100 mg or 200 mg/kg/mouse/day) and schedules of administration (7 to 18 days after leukemic cell injection) were first tested using the E2 unique subclone expressing or not expressing the WT1 protein (Table 1). Prolonged mouse survival (up to 150 days post-AML cell injection) was observed in 11% of E2/WT1-injected mice administered 3 daily doses of 200 mg/kg Ara-C 10 days after leukemic cell injection. This protocol was then applied using combinations of 2, 3 or 5 subclones. The best sustained mouse survival (42%) was observed when injecting the B11, C5 and E7 subclone mixture (B11/C5/E7) (Table 1 and Fig 3A). Over 31 mice injected, 13 were still alive 150 days after cell injection (Fig 3A). As previously described for the parental C1498 cell line [23], IV injection of these 3 *ZsGreen*-expressing subclones led to the development of AML in an average of 32 days (range from 29 to 38 days) (Fig 3A). Leukemic cells could be tracked due to ZsGreen protein expression, and their infiltration in the tissues was quantified by flow cytometry. Their frequencies ranged from 0.55±0.99% in the kidneys to 54.3±22.3% in the ovaries (Fig 3B) and varied from 1.2% to 29% in the peripheral blood (Fig 5A).

**Table 1:** Leukemic sub-clone(s) injections and cytarabine (Ara-C) administration protocols (doses and kinetics) for AML-bearing mice survival optimization.

Subclone(s)	Dose of chemotherapy per injection (kg/day/mouse)	Ara-C administration starting after the sub-clone(s) injection	AML onset (days mean)	Prolonged survival (surviving/injected mice and time of survival)
**Unique subclone (E2±WT1)**	without Ara-C	/	31	0/12
	100mg	3 injections starting at day 7		0/6
		3 injections starting at day 10		0/6
		5 injections starting at day 10		0/6
	200mg	3 injections starting at day 10	31	0/6 and **3/27 (11%)**
				**(150 days)**
				**(for E2/WT1)**
		3 injections starting at day 12		0/6
		3 injections starting at day 14		0/6
		3 injections starting at day 18		0/6
**2 subclones (E2 and F1±WT1)**	without Ara-C	/	31	0/6
	200mg	3 injections starting at day 10		0/6
**3 subclones (C5, B11 and E7)**	without Ara-C	/	32	0/6
	200mg	3 injections starting at day 10		**13/31 (42%)**
				**(150 days)**
**5 subclones (±WT1)**	without Ara-C	/	38	0/6
	200mg	3 injections starting at day 10		0/6
**3 subclones/WT1 (C5/WT1, B11/WT1 and E7/WT1)**	without Ara-C	/	38	**7/32 (21.8%)**
				**(136–142 days)**
	200mg	3 injections starting at day 10		**2/10 (20%)**
		5 injections starting at day 10		**8/14 (57.1%)**
				**(66 days) and**
				**11/18 (61.1%)**
				**(136–142 days)**
				**Total of 19/32**
				**(59.3%) (66 days)** **

Unique or different combinations of cytarabine-sensitive sub-clones expressing or not the WT1 antigen were injected into syngeneic and immune-competent mice. 10^5^ cells were injected by intravenous route per mouse. Chemotherapy was administered at various doses and intervals after the leukemic cell injection. The experiments were performed by groups of 6 to 10 mice per condition and repeated independently at least twice.

**, *p = 0*.*0008*, Log-rank test comparing AML disease-free mice following 5 doses of Ara-c or without treatment in B11/C5/E7/WT1-injected mice.

WT1 protein expression in the 3 subclones (B11/C5/E7/WT1) led to spontaneous survival for 21.8% of AML-injected mice (Table 1, Fig 3C). Administration of 5 doses of Ara-c (200 mg/kg/day/mouse) significantly improved mouse survival from 20% (3 doses) to 59.3% (19/32 surviving mice 66 days post cell injection, Table 1, Fig 3C). As observed in the absence of *Wt1* expression, AML-succumbing mice presented ZSGREEN^+^ leukemic cell infiltration in lymphoid and nonlymphoid organs, with frequencies ranging from 0.17±0.2% for the kidneys to 41.64±15.7% for the ovaries (Fig 3D). Thus, mouse survival could be achieved after chemotherapy using a mixture of the 3 subclones B11, C5 and E7 expressing or not expressing WT1.

### The C5 subclone is preeminent in the BM and PB of AML-bearing mice

**Fig 4:**
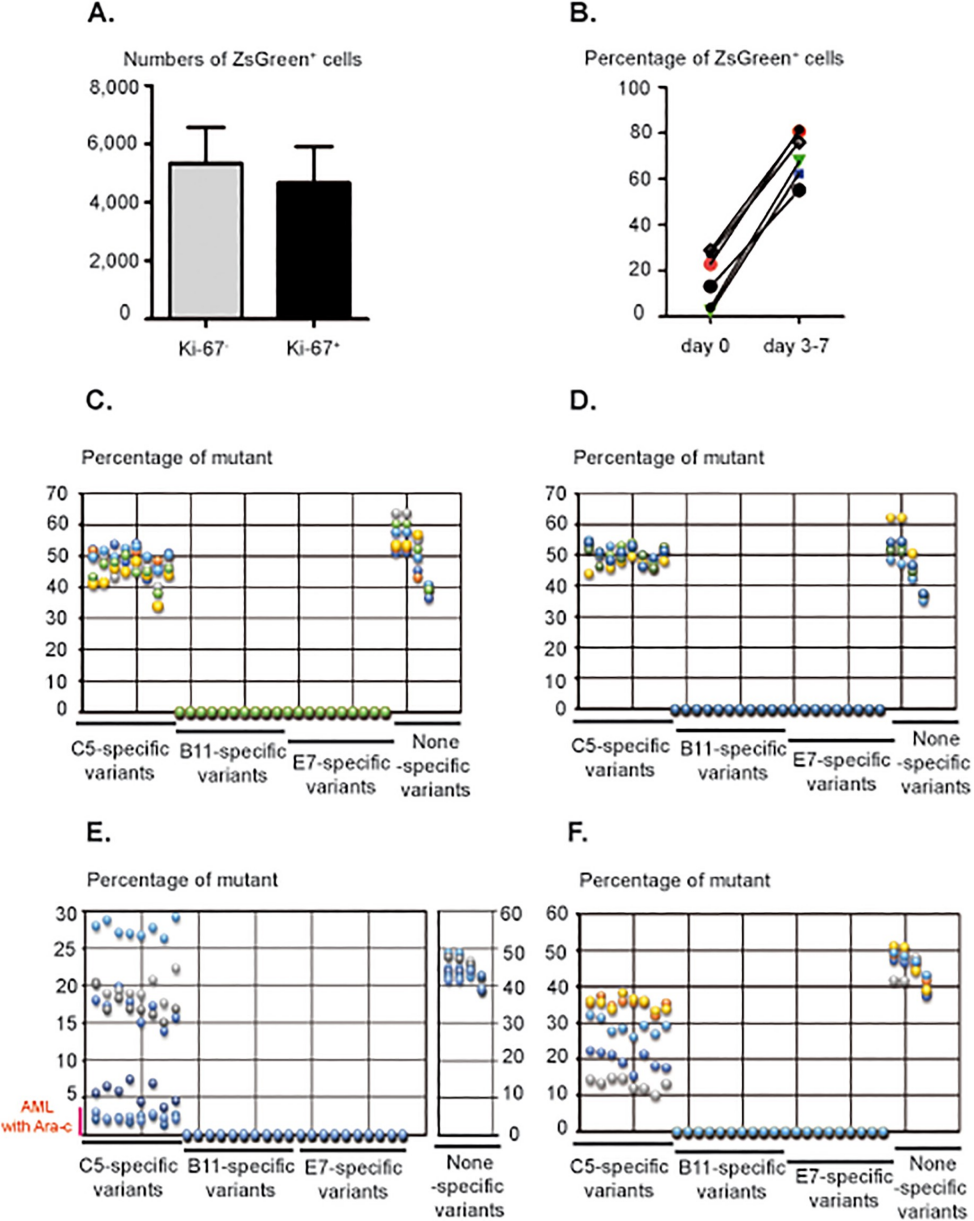
The C5 subclone predominates in the BM and PB of AML-bearing mice. (A) The numbers of leukemic Ki-67-negative and -positive cells in the BM of AML-succumbing mice expressing WT1 or not were determined by flow cytometry (n = 6). (B) *In vitro* culture of PB derived from AML-bearing mice (B11/C5/E7-injected mice) for 3 to 7 days (n = 6). Leukemic cell enrichment was followed by flow cytometry due to ZsGreen protein expression. (C) Percentages of subclone-specific variants determined by NGS in the PB of leukemic mice following B11/C5/E7 injection (n = 6) and (D) after inoculation with 3 WT1-expressing subclones (n = 5). (E) Frequencies of B11-, C5-, and E7-specific mutants in the BM of leukemic mice after the 3 *ZsGreen*-expressing subclone injections (n = 5) and Ara-c treatment (n = 2). (F) after B11/C5/E7/WT1 administration (n = 5). Non-specific variants represent mutations found of PB or BM of non-injected (control) mice, their frequencies were also estimated in the BM and PB of leukemic mice (treated or not with Ara-c).

As the quiescence of leukemic cells may favor resistance to chemotherapy and MRD, we assessed their proliferation in BM infiltrates of B11/C5/E7-injected mice (Fig 4A). Interestingly, we found comparable numbers of proliferating and nonproliferating ZSGREEN^+^ leukemic cells in BM (mean of 4,671±1,237 Ki67^+^ versus 5,329±1,237 Ki67^-^) (Fig 4A). To determine whether some subclones were more prone to quiescence, we sequenced whole BM tissues from both (expressing or not expressing WT1) AML mouse models. Peripheral blood samples were also cultured *in vitro* for 3 to 7 days to expand circulating ZSGREEN^+^ cells for sequencing (Fig 4B). The 3 different subclones could be distinguished due to some specific exomic mutations (Fig 4C–4F). The results revealed a prominent C5 subclone in the PB and BM of all AML-succumbing mice (Fig 4C and 4F). Similar results were also found in mice that succumbed to AML after Ara-c treatment (these mice presented lower ZSGREEN^+^ cell infiltration in the BM:~1.3 to 4.9%) (Fig 4E). Thus, taken together, these findings showed that C5 is the main subclone found in the BM and PB of mice during AML and after chemotherapy treatment.

### Detection of residual leukemic cells in surviving B11/C5/E7- and B11/C5/E7/WT1-injected mice

**Fig 5:**
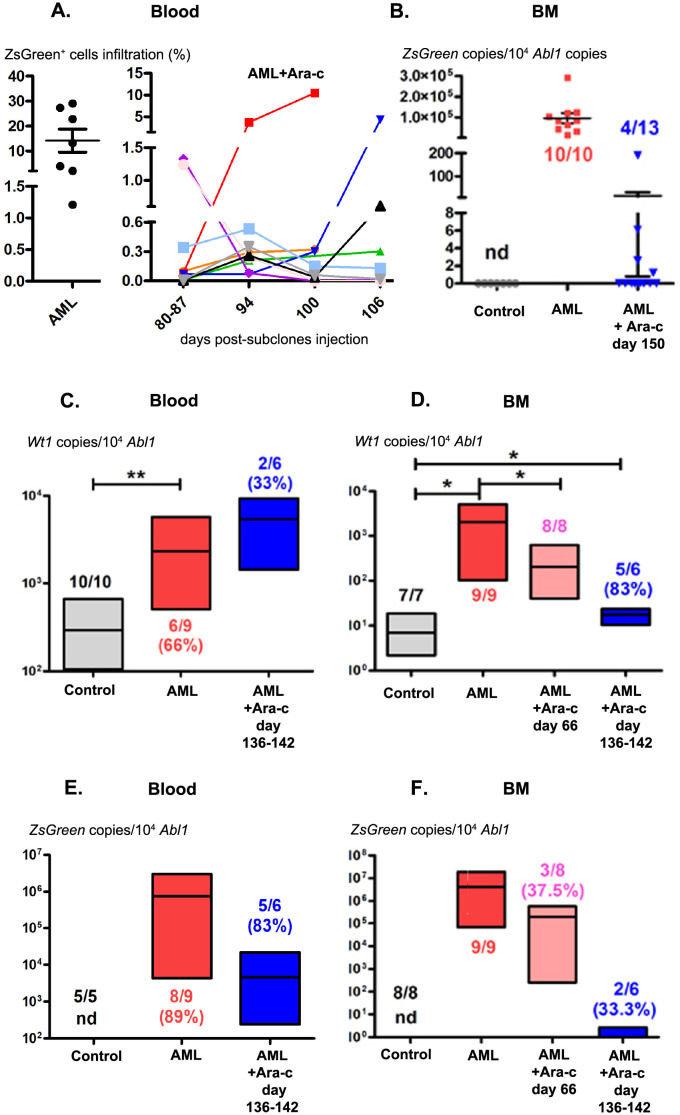
Detection of residual leukemic cells in AML-surviving mice after injection of B11/C5/E7 subclones expressing or not expressing WT1. (A) Percentages of ZSGREEN+ cells determined by flow cytometry in AML-succumbing (n = 7) and AML-surviving mice (n = 15) after B11/C5/E7 injection and Ara-c treatment, respectively. (B) RT-qPCR evaluation of *ZsGreen* copies and normalization per 10^4^ *Abl1* copies in the BM of control (n = 7), leukemic (n = 10) and disease-free mice after Ara-c treatment (n = 13). Determination of *Wt1* copies/10^4^ *Abl1* in peripheral blood (PB) (C) and BM (D) in control, AML-bearing, and AML-surviving mice with Ara-c treatment 66 and 136–142 days after B11/C5/E7/WT1 cell injection. Assessment of *ZsGreen* copies/10^4^ *Abl1* in PB (E) and BM (F) in control, AML-succumbing and live mice treated with Ara-c 66 and 136–142 days after B11/C5/E7/WT1 cell injection. The numbers of tested and positive mice are indicated in the graphs. nd = not detected. **, *p = 0*.*0053*, unpaired Student’s t-test comparing *Wt1* expression in the PB of AML-bearing and control mice. *, *p = 0*.*0285*, *p = 0*.*0157*, *p = 0*.*0129*, unpaired Student’s t-tests comparing *Wt1* expression in the BM of control versus leukemic mice and in the BM of tumor-bearing versus disease-free mice 66 and 136–142 days after cell injection, respectively.

As performed for AML-succumbing mice, leukemic cell infiltration was assessed by flow cytometry in peripheral blood and organs of AML-surviving mice. 9 out of 15 B11/C5/E7AML-surviving mice presented circulating ZSGREEN^+^ cells in the peripheral blood (Fig 3A). These cells could be tracked in each individual mouse from 80 to 106 days after subclonal injection (42 to 68 days after the AML control group succumbed) (Fig 5A). Their frequencies ranged from 0.1 to 1.33%, and 2 mice presenting higher percentages (10.48% and 4.33%, 100 and 106 days after cell injection, respectively) died of AML. The presence of circulating residual leukemic cells in the PB of B11/C5/E7/WT1 AML-surviving mice after Ara-c treatment was also confirmed through detection of *ZsGreen* and *Wt1* transcripts by RT-qPCR (Fig 5C and 5E). The expression of *ZsGreen* was tested in 6 treated mice 136 to 142 days after cell injection, 5 of which presented 4,691±4,307 copies/10^4^ *Abl1* (46.9% *ZsGreen*/*Abl1*) (~162-fold less than AML-succumbing mice, with a mean of 759,208±352,902 *ZsGreen* copies/10^4^ *Abl1* in 89% of mice) (Fig 5E). Similarly, 2 treated mice (including one mouse without *ZsGreen* expression) exhibited 5,393±3,955 *Wt1* copies/10^4^ *Abl1* (53.9%*Wt1*/*Abl1*) (~2-fold better than in AML-succumbing mice, with a mean of 2,323±807.8 *Wt1* copies/10^4^ *Abl1* in 66% mice) (Fig 5C).

The presence of leukemic cells in the BM was also assessed by RT-qPCR in both mouse models. A mean of 96,305±24,819 *ZsGreen* copies/10^4^ *Abl1* (963%*ZsGreen*/*Abl1*) were detected in all B11/C5/E7 AML-succumbing mouse BM (Fig 5B). In contrast, in surviving mice, *ZsGreen* copies could be detected in only 4 out of 13 animals (a mean of 50.2±46.9 copies/10^4^ *Abl1*, 0.*5% ZsGreen/Abl1*) 150 days after AML cell injection (Fig 5B). In B11/C5/E7/WT1 AML-surviving mice, *Wt1* and *ZsGreen* copies could also be detected 66 and 136–142 days after cell injection (Fig 5D and 5F). In the BM of these mice, the mean *Wt1* expression was decreased by ~1 log when assessed 66 days post cell injection compared to its expression in AML-succumbing mice (mean of 202.9±65.6 copies/10^4^ *Abl1* versus 2,020±721.3, respectively) (Fig 5D). The mean expression level was ~29-fold better than that in control mice (mean of 7.0±2.4–0.7% *Wt1/Abl1*). Interestingly, *ZsGreen* expression at the same time point was detected in only 37.5% of mice (mean of 195,161±194,710/10^4^ *Abl1*), suggesting a loss of its expression in the BM (Fig 5F). This observation was confirmed 136–142 days after cell injection as 83% of mice (5/6) exhibited significantly higher *Wt1* expression than that in control BM (mean of 17.5±2.3 copies/10^4^*Abl*), whereas *ZsGreen* transcripts were slightly detected (2 of 6 mice and mean of 0.8±0.5 copies/10^4^ *Abl1*). Thus, taken together, these observations suggested the presence of leukemic MRD in PB and BM after Ara-c treatment in both AML mouse models.

## Discussion

In this study, we generated a syngeneic and immune-competent mouse model of AML cell persistence expressing or not expressing the WT1 protein. We used different subclones with various intrinsic genetic and immunogenic properties [24], all of which demonstrated adequate sensitivity to Ara-c *in vitro*, and different combinations of cell injections, schedules and doses of chemotherapy were evaluated *in vivo*. Our main concerns were AML-bearing mouse survival kinetics and detection of residual leukemic cells over time. Bragado *et al*. suggested that 4 weeks in the mouse lifespan may be equivalent to 160 weeks (~3 years) in humans (~40-fold ratio, 2-year lifetime expectancy for mice versus 80 years for humans) [33]. Considering these criteria, the AML cell load can be followed in mice up to 7 weeks (49 days) post chemotherapy, which may correspond to the 60-month (260 weeks) period of relapse observed in patients treated for AML. In this sense, with or without expression of the WT1 antigen, we could detect residual leukemic cells in AML-surviving mouse PB and BM by flow cytometry and RT-qPCR from 56 days (8 weeks) up to 126–132 days (17–18 weeks) post-Ara-c treatment, mostly confirming their persistence.

We used cytarabine as the only chemotherapeutic agent at a dose of 200 mg/kg, which corresponds to 606 mg/m^2^, for 3 to 5 consecutive days [34]. This dose lies between the standard “7+3” regimen used in AML patients (3 days of anthracycline and 7 days of cytarabine 100mg/m^2^) and a high-dose regimen (1000 to 3000 mg/m^2^, every 12 hours, days 1,3,5,7) applied in AML patients. High-dose treatment does not confer benefits at induction but is widely used for consolidation in AML patients [2]. Such chemotherapy cycles (induction and consolidation) were difficult to define in leukemic mice, although we closely followed the leukemic cell burden. AML MRD is currently evaluated in patients through identification of a set of genetic markers (point mutations, fusion genes) at diagnosis in PB or BM and their detection by multiparameter flow cytometry and/or more sensitive quantitative molecular techniques (RT-qPCR, digital droplet PCR, NGS) after complete remission. The European Leukemia Net (ELN) MRD Working Group recommends the use of specific fusion genes (*RUNX1-RUNX1T1*, *CBFB-MYH11* and *PML-RAR*) or the mutated gene *NPM1* (one of the most frequent genetic aberrations in AML), which are strong predictors of relapses and can be detected with high sensitivity (up to 10^−6^) by RT-qPCR [28, 32]. In the absence of information about the fusion genes in our murine subclones, we assessed exomic mutations. No *Npm1* mutations were detected in our murine subclones, and we decided to use *Wt1* as a marker. Indeed, *WT1* overexpression can be assessed in patients when no other genetic markers are available; however, its detection must be performed using an ELN standardized assay [26, 28]. We followed these ELN guidelines to generate our *Wt1*-expressing AML MRD mouse model and to assess *Wt1* copies. In AML patients, the *WT1* expression background is defined as >250 *WT1* copies/10^4^ *ABL1* in BM and >50 *WT1* copies/10^4^ *ABL1* copies in PB. A >2-log decrease in BM after conventional chemotherapy was found to be predictive of a decreased risk of relapse [26]. *WT1* gene expression also represents a useful prognostic marker at CR before allo-HSCT for overall survival and the relapse risk [35]. *Wt1* expression thresholds in the BM and PB of mice were estimated to be 7±2.4 and 291.1±66 copies/10^4^ *Abl1*, respectively. Accordingly, we selected subclones expressing *Wt1* with the highest range and combined them (21,038±4,779 copies/10^4^ *Abl1* copies for the B11/C5/E7 mixture). We observed a 2-log decrease in the BM after cytarabine treatment in our mouse model 136–142 days after cell injection. As performed in AML patients’ post treatment, we also detected residual leukemic cells in mouse PB and BM. In contrast to AML patients, *Wt1*-expressing AML cells were more often detected in mouse BM than PB due to its lower detection threshold. However, *Wt1* monitoring by RT-qPCR in BM or PB after treatment could not predict relapse whatever their detection sensitivity. In patients, the MRD detection sensitivity by RT-qPCR does not always correlate with relapse. Indeed, it was shown in *NPM1*-mutated AML patients that MRD in PB can be more predictive of relapse even if the MRD detection sensitivity in BM is better [36]. In accordance with these findings, relapses in our mouse model after chemotherapy could be more easily predicted by increasing percentages of ZSGREEN^+^ cells in PB after detection by flow cytometry.

Interestingly, when we assessed for the detection of all subclones in PB, BM and other organs (lungs, liver and ovaries) in treated or not-treated leukemic mice, C5 was the major subclone. Although the 3 subclones presented a comparable sensitivity to Ara-c *in vitro*, C5-derived AML cells persisted after chemotherapy treatment. So far, this discrepancy cannot be explained by the ZsGreen fluorescent protein expression or its *in vivo* immunogenicity. Our previous study performed with these 3 subclones and their intraperitoneal injection in mice indicated that they were more immunogenic and resulted in lower lethality rates during AML development [24]. Moreover, the introduction of WT1 in these subclones, a valuable AML-associated antigen, also led to better AML-bearing mouse survival (~22%) without chemotherapy treatment. Further experiments will surely be performed to better delineate the roles of C5 subclone intrinsic properties (specific genomic alterations) and of the anti-WT1 specific immune response in leukemic cells’ elimination and/or persistence.

In contrast to humanized patient-derived xenograft (PDX) mouse models, which require 9 to 15 weeks to reconstitute a functional immune system and develop AML, we obtained leukemia MRD faster (2 weeks after cell injection), and our immune-competent model is highly reproducible and independent of patient sample availability.

The limits of this model reside in the detection thresholds of residual leukemic cells; although overexpressed, the *ZsGreen* and *Wt1* genes did not allow us to reach a sensitivity threshold lower than 10^−4^, as achieved with other genes in patients’ MRD follow-up (10^−6^ for mutated *NPM1*, for example). Thus, conclusions about AML cell persistence were difficult to draw in some surviving mice because of the sensitivity of our RT-qPCR assay (10^−4^ for *ZsGreen* and 10^−3^ for *Wt1*), but further experiments using digital RT-qPCR might help resolve this issue. Moreover, other factors, such as downregulation, loss or rearrangement of the *ZsGreen* and *Wt1* genes, may also interfere with the detection of residual AML cells. Recent isolation of PB ZSGREEN+ persistent cells will help to better characterize these leukemic cells (transcriptomic programs) and their genomic evolution. Hopefully, these studies should enable the development of future therapeutic strategies to eradicate chemo-resistant residual AML cells.

## Supporting information

S1 Fig WT1 expression does not alter *ZsGreen* transcripts and protein expression levels in stably pVITRO.1/Wt1-transfected subclones. (A) Microscopy immunofluorescent staining of the WT1 protein (pink) in a representative stably transfected subclone. Cells were harvested from cultures and centrifuged on slides for microscopy. Labeling of Myc-tag fused to the WT1 protein (see the Materials and Methods section) was performed on untransfected and pVITRO.1/Wt1-transfected cells using primary and secondary fluorescent antibodies. DAPI stain (blue) for cell nuclei was included in the mounting medium. For each microscopy image, an objective magnification of x20 was used. (B) Representative flow cytometry histogram of the ZsGreen protein expression in each subclone stably expressing *Wt1* gene. (C) RT-qPCR determination of *ZsGreen* expression (normalized to 10^4^ *Abl1* copies) in stably pVITRO.1/Wt1-transfected subclones and comparison with corresponding untransfected cells. The mean ± SEM from three independent experiments are shown.
(PDF)

S1 Raw images Raw images of the Western Blots. Detection of the WT1 (A) and actin proteins (B) in the different subclones. Cell lysates from subclones expressing (+WT1) or not the WT1 protein were loaded in the following order: E2, E2/WT1, C5, C5/WT1, F1, F1/WT1, B11, B11/WT1, E7 and E7/WT1 on SDS-PAGE gel before transfer to the membrane. A MagicMark^®^ XP Western Protein Standard (MM for Molecular Marker from 20 to 220 kiloDaltons) was loaded on each side of the gel. The images were captured using an ImageQuant^®^ LAS 4000. The Fig 2B was generated from these raw images.
(PDF)

S1 Raw data Raw data of targeted next generation sequencing in mice BM and PB. (TXT)
