# Supplementary figures and images for: Detection of residual and chemoresistant leukemic cells in an immune-competent mouse model of acute myeloid leukemia: Potential for unravelling their interactions with immunity
Source: PLoS One. 2022 Apr 29;17(4):e0267508. doi: 10.1371/journal.pone.0267508 (PMC9053800; doi:10.1371/journal.pone.0267508)

**A.**

untransfected  
subclone (mock)

pVITRO.1/Wt1-transfected  
subclone

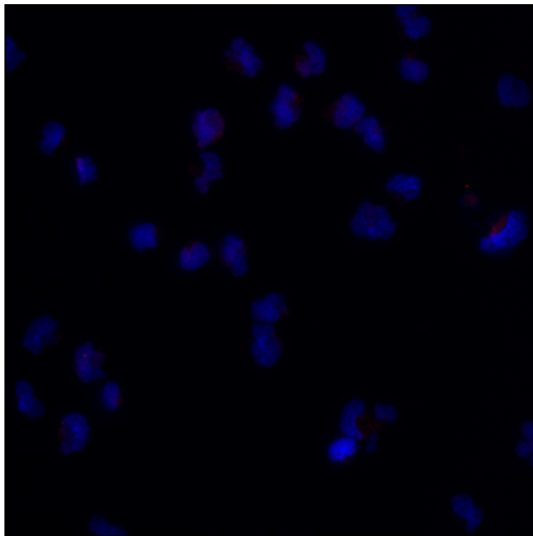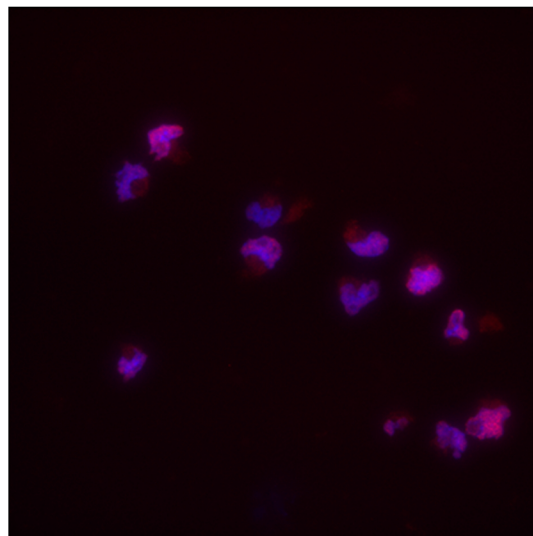

**B.**

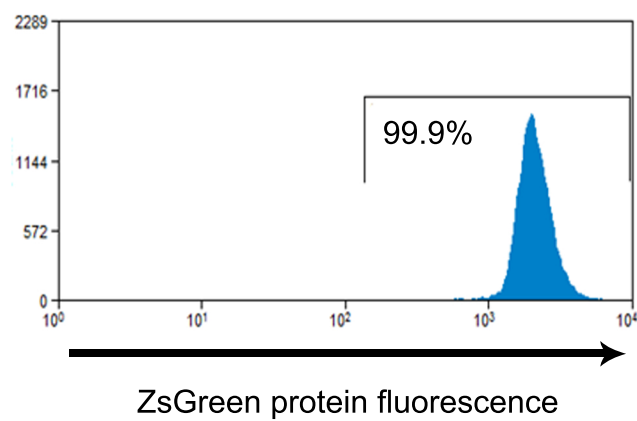

**C.**

*ZsGreen* copies/ $10^4$  *Abi1* copies

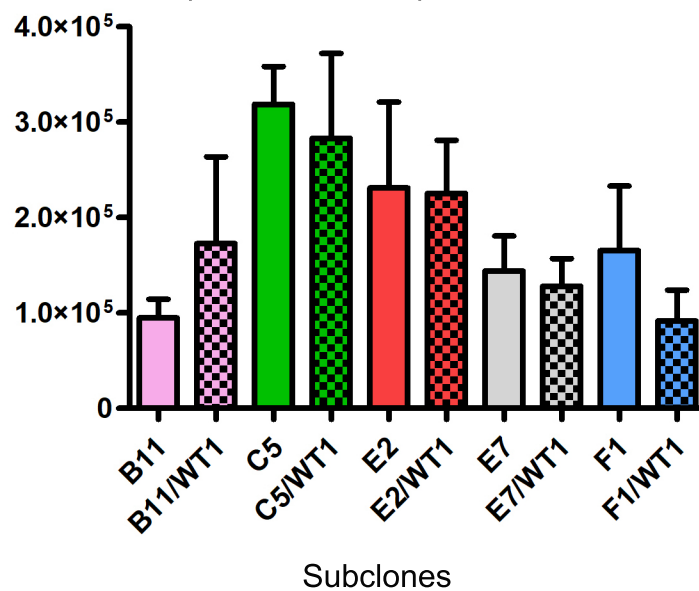

Supplement: S1 Fig — (A) Microscopy immunofluorescent staining of the WT1 protein (pink) in a representative stably transfected subclone. Cells were harvested from cultures and centrifuged on slides for microscopy. Labeling of Myc-tag fused to the WT1 protein (see the Materials and Methods section) was performed on untransfected and pVITRO.1/Wt1-transfected cells using primary and secondary fluorescent antibodies. DAPI stain (blue) for cell nuclei was included in the mounting medium. For each microscopy image, an objective magnification of x20 was used. (B) Representative flow cytometry histogram of the ZsGreen protein expression in each subclone stably expressing Wt1 gene. (C) RT-qPCR determination of ZsGreen expression (normalized to 104 Abl1 copies) in stably pVITRO.1/Wt1-transfected subclones and comparison with corresponding untransfected cells. The mean ± SEM from three independent experiments are shown. (PDF) [file pone.0267508.s001.pdf]
