# Supplementary material for: Detection of residual and chemoresistant leukemic cells in an immune-competent mouse model of acute myeloid leukemia: Potential for unravelling their interactions with immunity
Source: PLoS One. 2022 Apr 29;17(4):e0267508. doi: 10.1371/journal.pone.0267508 (PMC9053800; doi:10.1371/journal.pone.0267508)
Supplement: S1 Raw images — Detection of the WT1 (A) and actin proteins (B) in the different subclones. Cell lysates from subclones expressing (+WT1) or not the WT1 protein were loaded in the following order: E2, E2/WT1, C5, C5/WT1, F1, F1/WT1, B11, B11/WT1, E7 and E7/WT1 on SDS-PAGE gel before transfer to the membrane. A MagicMark® XP Western Protein Standard (MM for Molecular Marker from 20 to 220 kiloDaltons) was loaded on each side of the gel. The images were captured using an ImageQuant® LAS 4000. The Fig 2B was generated from these raw images. (PDF) [file pone.0267508.s002.pdf]

A.

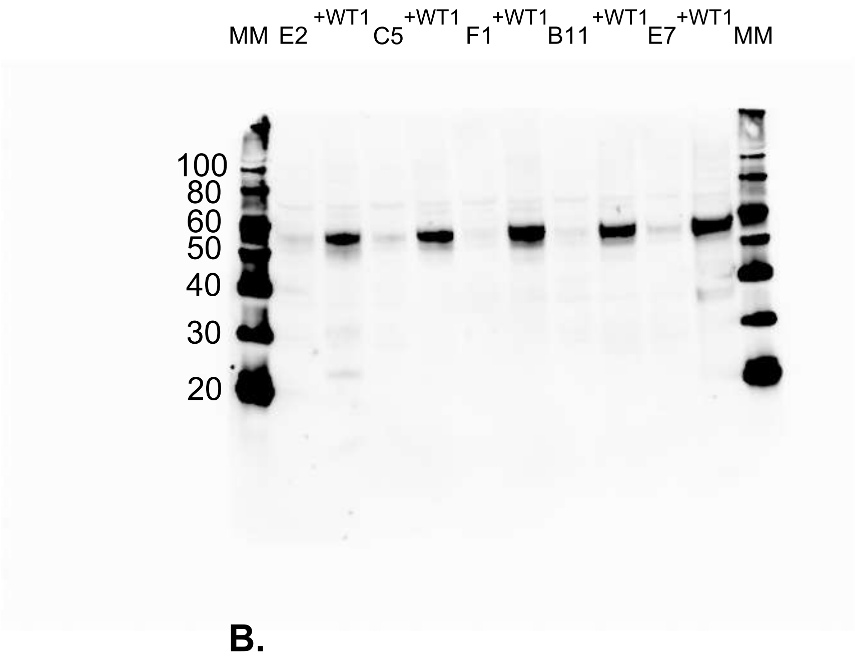
MM E2 +WT1 C5 +WT1 F1 +WT1 B11 +WT1 E7 +WT1 MM
100
80
60
50
40
30
20

B.

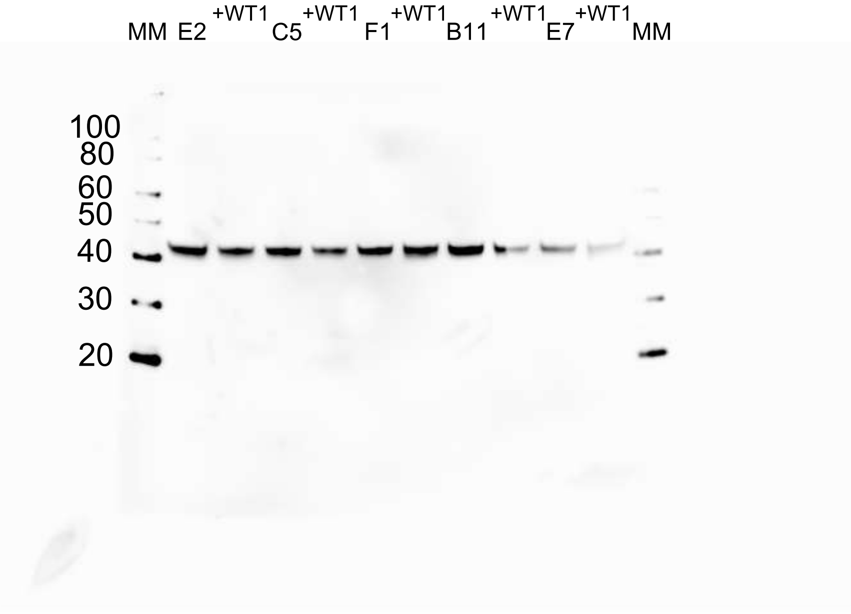
MM E2 +WT1 C5 +WT1 F1 +WT1 B11 +WT1 E7 +WT1 MM
100
80
60
50
40
30
20
